# Dual application of β-sitosterol and biochar reduces copper toxicity in bamboo via improved redox homeostasis

**DOI:** 10.3389/fpls.2025.1554519

**Published:** 2025-08-19

**Authors:** Abolghassem Emamverdian, Xinyu Lyu, Necla Pehlivan, Li Zhang, Haider Sultan, Yang Li, Meisam Zargar

**Affiliations:** ^1^ Co-Innovation Center for Sustainable Forestry in Southern China,Nanjing Forestry University, Nanjing, China; ^2^ Bamboo Research Institute, Nanjing Forestry University, Nanjing, China; ^3^ Department of Biology, Recep Tayyip Erdogan University, Rize, Türkiye; ^4^ School of Breeding and Multiplication (Sanya Institute of Breeding and Multiplication), Hainan University, Sanya, China; ^5^ Department of Mathematics and Statistics, Florida Atlantic University, Boca Raton, FL, United States; ^6^ Department of Agrobiotechnology, Institute of Agriculture, RUDN University, Moscow, Russia

**Keywords:** bamboo species, biochar application, β-sitosterol, plant stress tolerance, heavy metals

## Abstract

**Introduction:**

Heavy metal pollution threatens ecosystems and agriculture, necessitating affordable solutions.

**Methods:**

We evaluated the combined effect of β-sitosterol (Bs, 100 mg L^-1^) and eucalyptus biochar (Eb, 10%) on bamboo (*Sasa kongosanensis* f. *aureo-striatus*) under copper stress (100 and 200 mg L^-1^ Cu).

**Results and discussion:**

Elevated Cu induced oxidative stress via reactive oxygen species (ROS) and methylglyoxal (MG) impairing photosynthesis, nutrient uptake, and growth. Bs and Eb, individually or combined, enhanced antioxidant activity (SOD, CAT, POD, PAL), glyoxalase cycle efficiency, and osmolyte accumulation (proline, glycine betaine), mitigating oxidative damage. The treatments improved photosynthetic pigments, gas exchange, and water retention while reducing Cu translocation and bioaccumulation. Combined Bs+Eb most effectively lowered Cu levels in roots (36–45%), stems (35–38%), and leaves (24–51%) compared to controls. Nutrient uptake (Fe, Mg, Mn, K, P, Ca) was increased by 12–44% with Eb and 7–25% with Bs alone, yet synergistically by 87–190% with Bs+Eb. Biomass and shoot length were improved by 26–54% under Cu stress. The dual application also reduced electrolyte leakage (41–66%) and MG content (12–19%) while boosting non-enzymatic antioxidants (GSH, AsA) by 67–139%. These results demonstrate that Bs and Eb jointly enhance bamboo tolerance against Cu by improving redox homeostasis, nutrient retention, and stress resilience. This approach offers a sustainable strategy for phytoremediation and soil restoration in Cu-contaminated environments.

## 1 Introduction

Heavy metals (HMs) cause significant physiological and biochemical alterations in plants, leading to altered nutrient assimilation, senescence, water balance, low biomass, growth inhibition, photosynthesis limitation, chlorosis, and plant death (Singh et al., 2016; Pehlivan et al., 2023; Emamverdian et al., 2025a). On the other hand, the destructive impact of HMs on plants increases the risk of long-term exposure and potential health risks for both society and the ecosystem with their persistence in nature (Yang et al., 2005; Emamverdian et al., 2018a, 2021a, 2021b). Among these metals, copper (Cu) is crucial for plant growth (Tapiero et al., 2003), which is bound with amino acids that are vital components in antioxidant processes (Prashanth et al., 2015). Nevertheless, the primary characteristics of vital nutrients are connected to regulatory mechanisms that can sustain the elements at nutritional levels, hence averting detrimental effects in excessive concentrations (Hu et al., 2019). Furthermore, Cu is one of China’s most abundant toxic metals in agriculture and forest land soils (Zhang et al., 2015; Emamverdian et al., 2022a). The threshold for Cu toxicity in the soil is up to 150 ppm in silty-clay/clay soils and 50 ppm in sandy soils (Keita et al., 2023). Excessive Cu induces reactive oxygen species (ROS) overproduction and methylglyoxal (MG) in plants, leading to increased oxidative stress by enhancing lipid peroxidation in the cell membranes (La Torre et al., 2018; Miotto et al., 2014). It can also impact the fundamental process of plant photosynthesis (Ambrosini et al., 2018), leading to plant growth and development restrictions. Thus, ROS and MG are among the primary compounds produced in plant stress conditions, leading to disorder in plant metabolism (Yadav et al., 2005a), which was previously addressed along with their role in signaling pathways that contribute to stress adaptations as natural responsers (Namdjoyan and Kermanian, 2013; Mousavi et al., 2020). Plants develop mechanisms to counteract ROS compounds, using both antioxidant enzyme activity and non-enzymatic processes. Antioxidant enzyme activities include peroxidase (POD), superoxide dismutase (SOD), ascorbate peroxidase (APX), glutathione reductase (GR), catalase (CAT), dehydroascorbate reductase (DHAR), and mono dehydroascorbate reductase (MDHAR). Non-enzymatic antioxidants include tocopherols, phenolic compounds, ascorbic acid (AsA), and glutathione (GSH). Plants modulate their antioxidant defense networks in response to Cu exposure by enhancing the activities of both enzymatic components, such as SOD and CAT, and non-enzymatic components, including glutathione and ascorbate (Emamverdian et al., 2025b). Additionally, the glyoxalase system (Gly I and Gly II) serves as a primary defense system to scavenge MG. This system and antioxidants form the frontline of plant defense mechanisms, ultimately improving plant tolerance. In addition, plants set up an intricate defense mechanism with detoxification processes and sequestration strategies involving Cu export to decrease Cu stress through lowering internal levels of Cu. Among the Cu toxicity defenses by plants are the two major active phytochelatins, which bind the Cu ions into less toxic compounds stored within the vacuoles, and metallothioneins, which enhance the detoxification of Cu through their structure rich in cysteine to maintain homeostasis. By the compartmentalization process, plants protect their cytoplasm from Cu by translocating excessive metals into vacuoles and immobilizing them through cell wall binding. They also respond by using the root barriers via increasing the density of casparian strip cells and increasing levels of suberin to reduce the entry of Cu (Chen et al., 2022; Xu et al., 2024; Wang et al., 2024).

Recently, there has been a concentrated effort in research to utilize sustainable techniques and strategies to eliminate environmental and cellular toxicity (Yakhin et al., 2017; Carillo, 2018). Researchers put effort into finding ways that enhance the plants’ ability to resist contamination by HMs. They apply two main approaches in phytoremediation through plant-based processes, extracting or stabilizing contaminants (Aryal, 2024) and applying genetic modification to increase plant tolerance to HMs, as discussed by Iqbal B. et al. (2024), in conjunction with biochar applications to the soil that retains HMs, preventing release to the environment (Sarraf et al., 2024), and plant growth-promoting rhizobacteria (beneficial microbes) that help plants survive in a contaminated environment (Iqbal M. Z. et al., 2024; Vocciante et al., 2022). Phytosterols, which include brassinosteroids (BRs) and sterols, are also recognized as plant growth regulators that can mitigate the detrimental effects of HMs. Additionally, they promote plant growth and development (Vriet et al., 2012; Ines et al., 2019).

Biochar (BC) is a carbon-based substance that helps enhance the levels of mineral nutrients, fundamental cations, and water quality in soils. This, in turn, improves the general integrity and fertility of the soil, leading to sustained plant production (Praveen et al., 2022; Singh Mavi et al., 2018; Sun et al., 2018). Biochar can also influence soil’s overall pore volume and compactness, enhancing its ability to store carbon over a long period (Pehlivan and Wang, 2022; Khademalrasoul et al., 2014; Chen et al., 2019). Additionally, these materials can regulate the seasonal and daily temperatures of soil, thus altering the soil’s thermal dynamics (Li et al., 2020; Xiu et al., 2021). Research has documented that biochar positively impacts plants’ biochemical and morphological characteristics when subjected to challenging events, either long or short-term (Hammer et al., 2015; Winarso et al., 2021). Different studies confirm that these valuable substances deliver multiple advantages that boost crop production through enhanced soil fertility and increased nutrient accessibility. The functional architecture of biochar also enables enhanced nutrient availability through two strategies, which include reduced nutrient leaching processes and improved fertilizer effectiveness, and they act as a binding agent to minimize the hazardous effects of HMs and organic pollutants by which they become less bioavailable to plants (Khan et al., 2024; Murtaza et al., 2024; Al Masud et al., 2023).

Bamboo, a unique plant that overgrows and remains green throughout the year, is a member of the Gramineae (*Poaceae family*). Categorized as *Bambusoideae*, it is a perennial woody plant known for its ecologically beneficial properties and is a source of cost-effective forestry products (Jin et al., 2016; Ahmad et al., 2021). Bamboo is acknowledged as a non-timber forest resource that has been utilized for several purposes (Emamverdian et al., 2023c). According to Yan et al. (2015), nearly 6 million hectares of forest in China have been enveloped by bamboo plants, which exceed 30 million hectares of forested land worldwide. There are around 500 bamboo species belonging to 48 genera in China. These species are of significant economic value to the local population, especially in Southern China (Huang et al., 2015). *Pleioblastus kongosanensis f.aureo-striatus* is a decorative bamboo species with natural antioxidant properties characterized by its green leaves adorned with yellow stripes. In various metropolitan regions of Eastern and Southern China, such as Jiangsu province, *Pleioblastus kongosanensis* has been utilized for its aesthetic and landscaping purposes. Furthermore, it is an indicator for predicting soil and air pollution in these areas (Huang et al., 2015). Consequently, the accumulation of HM toxicity, particularly excess Cu, poses a significant challenge and impediment to the growth of bamboo. Therefore, exploring the use of cost-effective and natural materials to mitigate Cu contamination in bamboo plants, soil, and the environment is imperative. This has led to using β-sitosterol (Bs) combined with eucalyptus biochar (Eb) in this research. Limited data exists regarding the potential effects of β-sitosterol and eucalyptus biochar on plants.

β-Sitosterol functions naturally as a phytosterol. It also acts as a crucial mechanism against Cu-induced oxidative stress through ROS scavenging and increased activity of SOD and CAT enzymes (Rossi and Huang, 2022; Alharbi et al., 2023). The protective system preserves cell structures and limits harmful reactions between oxygen and plant lipids, which results in increased tolerability of HMs in plants. By integrating Eb treatment with Bs application, agricultural systems obtain united support for plant development through soil remediation of Cu-contaminated areas. By simultaneously improving soil health and plant tolerance in the face of HM toxicity, this integrated strategy shows promise for sustainable agriculture. Specifically, Bs and Eb play synergistic roles in enhancing plant tolerance to Cu stress through distinct yet complementary mechanisms. Consequently, using these two substances separately or in combination can yield favorable results in eliminating toxins from plants and soil. Because β-Sitosterol (1) acts as a signaling molecule that up-regulates the plant’s antioxidant defense system, (2) boosts the glyoxalase cycle (Gly I and Gly II), which detoxifies MG, a cytotoxic byproduct of Cu-induced stress, (3) promotes the accumulation of osmolytes such as proline and glycine betaine (GB), which stabilize cellular structures, protect enzymes, and maintain osmotic balance under stress conditions. Eucalyptus Biochar, on the other hand, (1) reduces Cu bioavailability in the soil by adsorbing Cu ions onto its porous surface, thereby limiting their uptake by plant roots, (2) improves soil structure, enhancing water retention and nutrient availability. When applied together, Bs and Eb create a dual protective mechanism. Bs strengthens the plant’s internal defense systems, while Eb reduces external Cu exposure. This combination enhances photosynthetic efficiency, maintains water balance, and ensures normal growth by minimizing Cu-induced oxidative stress and improving mineral uptake. Thus, their combined application provides a robust strategy for improving plant tolerance to heavy metal toxicity (Alharbi et al., 2023). This study is particularly novel because it investigates the synergistic effects of β-sitosterol and eucalyptus biochar in improving HM tolerance, specifically in bamboo, a plant that has received limited attention in this context. While both treatments have been studied independently, their combined application offers a unique approach to enhancing phytoremediation potential in bamboo under Cu stress. The exploration of these interactions not only provides new insights into the mechanisms of plant adaptation to metal toxicity but also contributes to the development of innovative strategies for sustainable agriculture, particularly in regions severely affected by HM pollution.

This study aims to address the following question: How do the combined applications of β-sitosterol and eucalyptus biochar affect the tolerance of bamboo (*Pleioblastus kongosanensis f. aureo-striatus*) to copper toxicity? The study hypothesized that the combined application of β-sitosterol and eucalyptus biochar would enhance bamboo tolerance to Cu stress by improving nutrient uptake, water retention, and antioxidant activity, thereby mitigating oxidative damage and promoting growth. Thus, the objective of this study was to utilize β-sitosterol and eucalyptus biochar to assess the reduction of the adverse effects of Cu accumulation on bamboo plant species while examining the physiological and biochemical mechanisms involved in this process which represents the first attempt to investigate the impact of these substances on bamboo plants, shedding light on key mechanisms involved in alleviating Cu toxicity in plants.

## 2 Materials and methods

The Bamboo Garden of Bamboo Research Institute (Nanjing Forestry University, Nanjing, China) supplied one-year-old bamboo plants, specifically *Sasa kongosanensis f. aureo-striatusor*. Plants were cultivated in pots (30cm X 30) with a completely random design (CRD containing five plants. Then, the pots were relocated to a controlled greenhouse where they were exposed to a photoperiod of 16 h of light and 8 h of dark, along with a humidity ratio ranging from 65% to 75%, for a duration of 60 days. The growth medium contained a mixture of 3 L per pot, which consisted of coco peat and perlite at a ratio of 1:2 for 20 treatments, each replicated 4 times, as presented in **Table 1**. Each treatment contained 4 biological replicates with 5 plants per replicate (n=20 plants/treatment). Three technical replicates ensured assay precision (CV <10% for all analyses). The pots were watered 5X throughout the trial period using 250 ml solution. A solution containing 100 mg L^-1^ of β-sitosterol (Bs) was manually sprayed 5X every 9 days using a hand spray. In addition, once the pots were filled with the growth medium, Eucalyptus biochar (Eb) was added at a concentration of 10% by weight (120 g per pot) and evenly mixed into the soil. The bamboo pots were supplied with a 400 mL nutrient mix (Emamverdian et al., 2023c) that contained K_2_O as a source of potassium sulfate, P_2_O_5_ as a source of calcium phosphate, and (NH_4_)_2_SO_4_ as a nitrogen fertilizer. The leaves, stems, and roots were harvested 2 weeks after the most recent application of β-sitosterol (Bs) spray to determine biochemical and physiological variables. Each study used four biological replicates for each treatment. We used Cu concentrations of 100 and 200 mg L^-1^ to cause stress in plants because these levels match the levels encountered in contaminated areas. We basically chose the solution concentrations based on two ends of the tolerance spectrum of bamboo species. In our previous studies, we showed these concentrations created effective plant growth disruption, reduced chlorophyll content, and increased oxidative stress markers that made them suitable for Cu toxicity research and mitigation tests. Therefore, applied concentrations simulate real-life stress conditions to determine how Bs with Eb reduced damage from Cu stress.

**Table 1:** The experimental design.

Treatments	Concentrations
Control	0
Cu	100 mg L^-1^ Cu
Cu	200 mg L^-1^ Cu
Bs	100 mg L^-1^ Bs
Bs + Cu	100 mg L^-1^ Bs + 100 mg L^-1^ Cu
Bs + Cu	100 mg L-1 Bs + 200 mg L^-1^ Cu
Eb	10% Eb
Eb+Cu	10% Eb + 100 mg L^-1^ Cu
Eb+Cu	10% Eb + 200 mg L^-1^ Cu
Bs+ Eb	100 mg L^-1^ Bs + 10% Eb
Bs+Eb + Cu	100 mg L^-1^ Bs +10% Eb + 100 mg L^-1^ Cu
Bs+Eb + Cu	100 mg L^-1^ Bs +10% Eb + 200 mg L^-1^ Cu

### 2.1 Determination of Cu and nutritional value (mineral content) in bamboo plants

The Ismail et al., 2022 technique was used to assess Cu and nutrient levels (N, P, K, Ca, Mn, Fe, and Mg). Five g dry samples were added to nitric acid (5 ml) and incubated at 30°C overnight. The samples were then dried at 95°C (Khosropour et al., 2019).

### 2.2 Determination of photosynthetic pigments and gas exchange

The fresh *Pleioblastus kongosanensis* leaves (0.2 g) were used to evaluate photosynthetic pigments. The absorbance at 480, 645, and 663 nm for chlorophyll a, b, and carotenoids was recorded on a spectrophotometer (Jenway, Japan) (Arnon, 1949). Also, an infrared gas analyzer (IRGA, TPS-2, USA) was used to determine the gas exchange parameters 40 days after sowing between ZT3 and ZT5 (Sun et al., 2018).

### 2.3 Determination of biochemical attributes and electrolyte leakage

To find out how much H_2_O_2_ existed in the samples, spectroscopic measurements were used. In accordance with Aftab et al. (2011), the absorbance of the reaction mixture was measured at a certain wavelength, and the final concentration of H_2_O_2_ was determined by using the extinction coefficient of 0.28 μM^-1^ cm^-1^. In micromoles per gram of fresh weight (μM g^-1^ FW). The superoxide radicals (O_2_•−) were determined by a reference curve created with nitrogen dioxide radicals (NO_2_•) as per Li et al. (2010). The lipoperoxidation index in plants was measured via MDA amount (Djanaguiraman et al., 2010). The content was obtained by calculating the molar extinction coefficient (155 mM^−1^ cm^−1^), expressed as μM MDA g^−1^ FW. Electrolyte leakage (EL) was determined using the protocol of Valentovic et al. (2006), calculated by measurement of primary electrical conductivity (EC_1_) and the last value of electrical conductivity (EC_2_) using the below formula:


EL(%)=(EC1/EC2)×100

### 2.4 Determination of plant osmolyte (soluble carbohydrate and proline) and GB contents

The proline content was quantified by the Zhang technique (Zhang et al., 2012), which involved multiple adjustments. The optical density (OD) was measured at 520 nm on the spectrophotometer (mg g^−1^ FW). The concentration of soluble carbohydrates was obtained using the enthrone colorimetry described by Zhang et al. (2012). Glycine betaine (GB) concentration was determined based on Grieve and Grattan (1983). The absorbance (at 365 nm) was measured using a baseline curve to derive the GB.

### 2.5 Measurement of relative water contents and the leaf water potential

The leaf water potential (LWP) was determined by a psychrometer (between ZT3 and ZT5) in the daytime. The relative water content (RWC%) and water content (WC%) were estimated by Kaya et al. (2013) and Fernandez-Ballester et al. (1998) by following the below formula for the calculation of the final RWC:


RWC(%)=[(FM−DM)/(SM−DM)]×100

FM (Fresh Mass)
DM (Dry Mass)
SM (Saturated Mass)

For these measurements, four biological replicates per treatment were used.

### 2.6 Determination of non-enzymatic antioxidants

Reduced glutathione (GSH) and oxidized glutathione (GSSG) were measured on plant leaves using Anderson’s (1985) protocol. Ascorbic acid (AsA) was measured using 2 mL of 5% TCA on 0.2 g of fresh leaves according to the methods adopted by Jagota and Dani (1982), where the AsA content was determined by 760 nm absorbance readings.

### 2.7 Determination of antioxidant enzyme activities and glyoxalase cycle efficiency

The superoxide dismutase (SOD - EC1.15.1.1) was assessed by Senthilkumar et al. (2021). This involved the photo-reduction rate calculations of nitroblue tetrazolium (NBT) to determine the final OD by recording 560 nm readings. The activity of peroxidase (POD - EC.1.11.1.6) was measured using the procedures described by Zhang et al. (2012). The molar extinction coefficient was employed at a value of 26.6 mM^−1^ cm^−1^, and the OD was determined at 436 nm. The Catalase activity (CAT-1.11.1.7) was read at 240 nm as the rate of H_2_O_2_ degradation (mg^−1^ protein) (Aebi, 1984) using 39.4 mM^−1^ cm^−1^ coefficient (1 unit = 1 mM of H_2_O_2_ reduction min^−1^). The PAL activity represented as U mg^−1^ of protein was estimated using the procedures described by Berner et al. (2006). The Glyoxalase (Gly I) activity at 240 nm for a duration of 1 min. was detected by Hasanuzzaman et al., 2011. The Gly II activity was calculated using the approach established by Principato et al. (1987). The protein extract was analyzed at 412 nm for a minute. The Methylglyoxal (MG) data was gathered by following the procedures outlined by Yadav et al. (2005b). The absorbance of the derived MG was measured by monitoring the range of 200–500 nm for 17 cycles, with a 1-min. intervals between each cycle.

### 2.8 Determination of plant biomass and plant growth

The shoot lengths were measured, and the shoots and roots of plants were washed and cleaned at the end of the experiments. The samples were placed in a vacuum-drying oven (DZF-6090) to remove the surface water, which was kept at 120°C for 30 min. and maintained at 76°C for 48 h. The dried samples (root and shoots) and shoot lengths were taken.

### 2.9 Translocation factor, bioaccumulation factor, and tolerance index

To assess the potential of bamboo plants for phytoremediation and phytoextraction efficiency under Cu treatment, translocation factor (TF), bioaccumulation factor (BAF), and tolerance index (TI) were calculated using the following formulas (Souri and Karimi, 2017):


(1) TI of the shoot=(shoot dry weight)/(dry weight of the control)

(2) TI of the root=(root dry weight)/(dry weight of the control)

(3) BAF=(Cu levels in the plant root or shoot)/(Cu levels in the medium)

(4) TF=(the levels of Cu in the stem and leaves)/(levels of Cu in the roots)

### 2.10 Statistics

This study utilized a completely randomized design (CRD) with four 4 independent biological replicates (n=5 plants each). Data were analyzed using R 4.3.1 (lme4 package) via two-way factorial ANOVA with Tukey’s HSD *post-hoc* tests (α=0.05). Effect sizes were reported as partial η² for significant terms. The data were also analyzed using Analysis of Variance (ANOVA). Mean differences were demonstrated, and Duncan’s *post hoc* test was used at a significance level of p < 0.05. Normality was confirmed using Shapiro-Wilk (W >0.92, p>0.10) and Anderson-Darling (A² <0.35, p>0.08) tests, with homogeneity of variance verified via Levene’s test (F(19,60)=1.12, p=0.35). Outliers (>3 SDs from the mean) were retained after verifying they represented biological variation (QQ plots showed no systematic deviations).

## 3 Results

### 3.1 Copper content in bamboo plant organs (leaves, shoots, and root)

**Figure 1:**
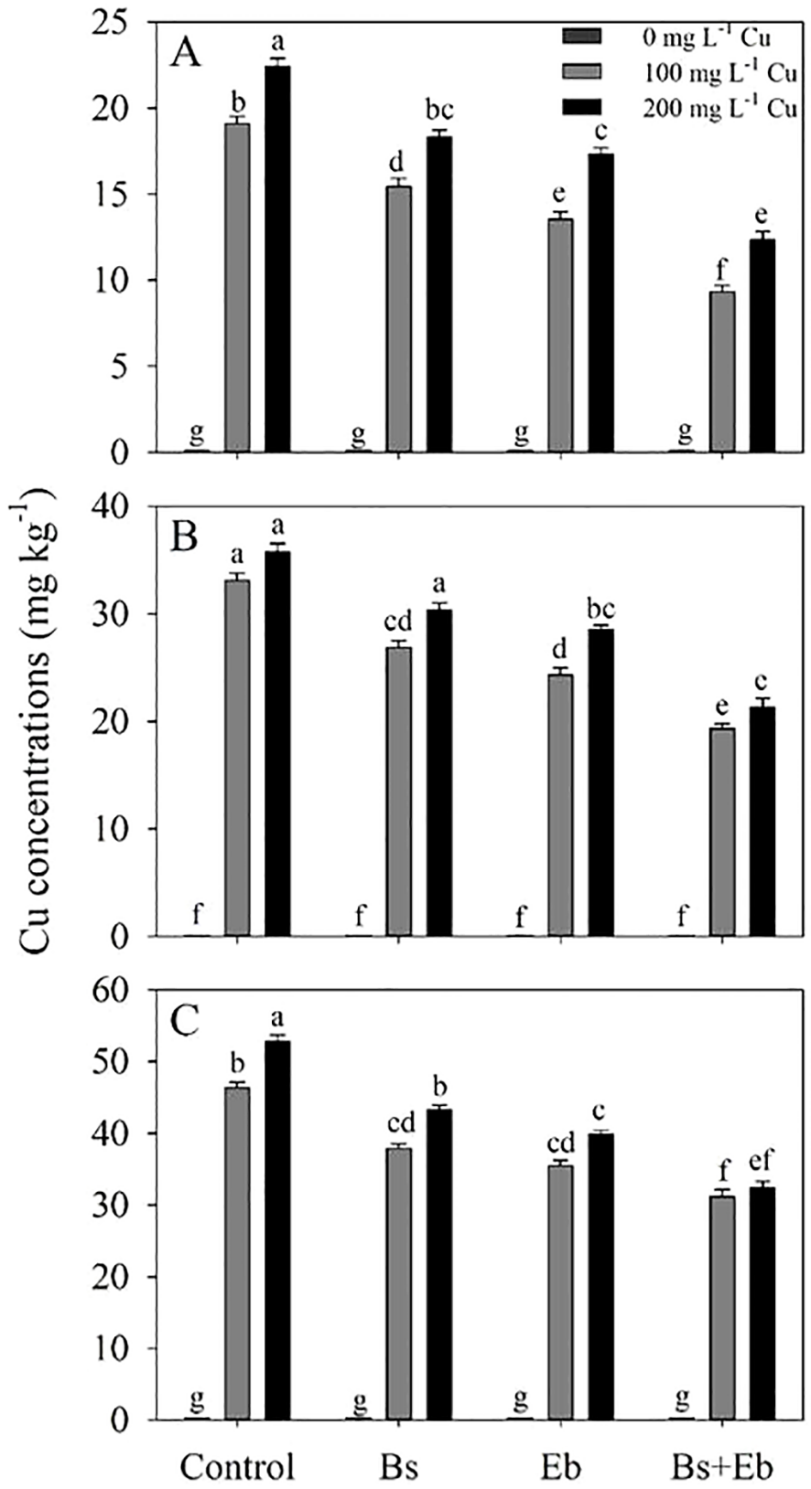
The impact of β-sitosterol and eucalyptus biochar on bamboo *(Pleioblastus kongosanensis* L.) Cu content in leaves **(A)**, stem **(B)**, and roots **(C)** under 100 and 200 mg L^-1^ copper in four replications. Control 0 mg L^-1^ Cu, Bs (β-sitosterol, 100 mg L^-1^), Eb (eucalyptus biochar, %10). The lowercase letters (a, b, c, etc.) demonstrated significant differences among the treatments (Duncan, p < 0.05).

Cu levels in various sections of bamboo plants indicated that the combination of β-sitosterol (Bs) and eucalyptus biochar (Eb) led to a significant decrease in the available Cu amount. The combination of Bs+Eb+100 mg L^-1^ Cu reduced Cu levels by 51%, 35%, and 24% compared to the control treatment. Further, the combination of Bs + Eb reduced 200 mg L^-1^ Cu by 45%, 38%, and 36% compared to the controls. The effect of Eb on Cu reduction was more significant than that of Bs alone. Ultimately, both Eb and Bs effectively decreased Cu levels compared to respective controls (**Figure 1**).

### 3.2 Mineral content and nutritional value in plants

**Figure 2:**
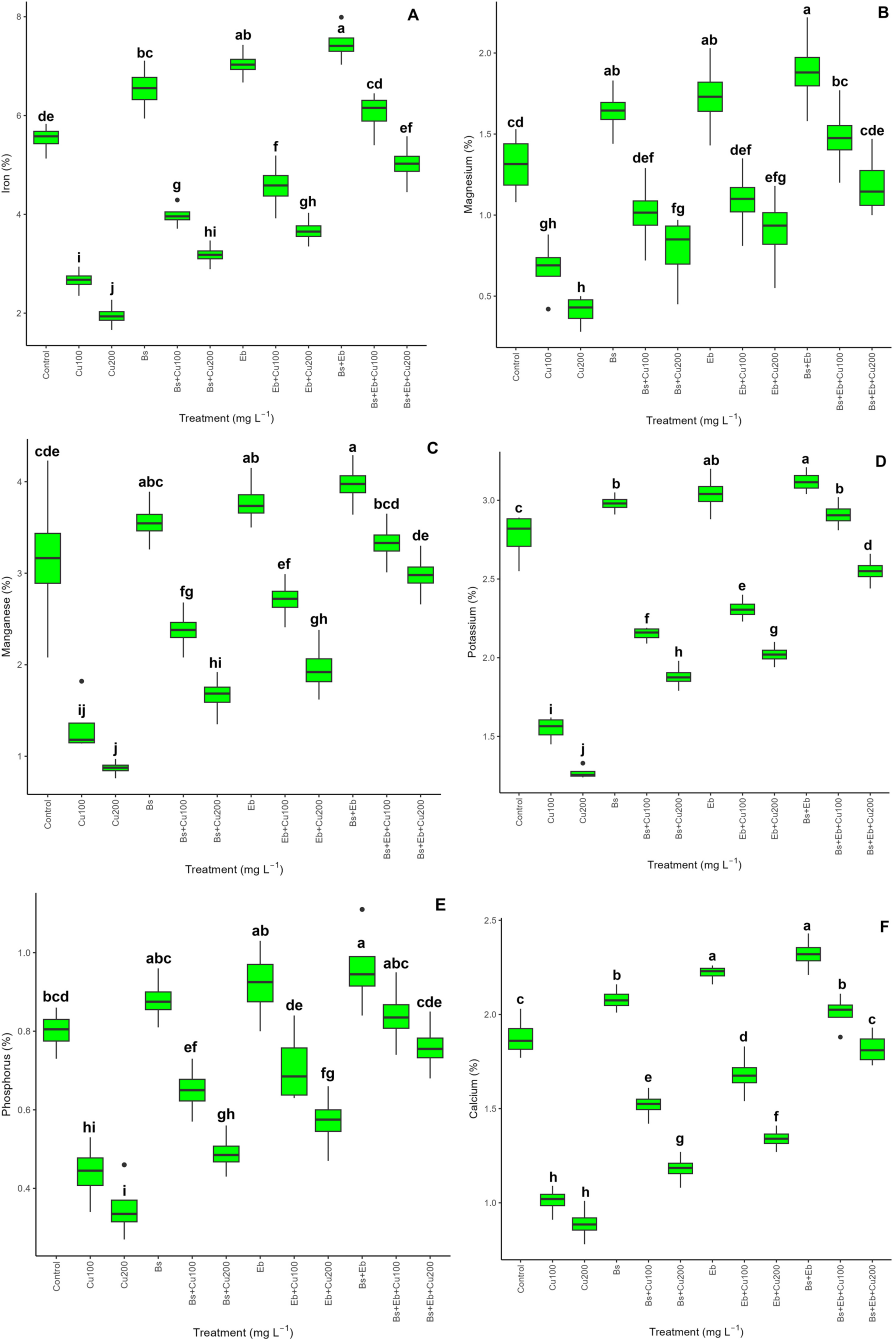
The impact of β-sitosterol and eucalyptus biochar on bamboo’s (*Pleioblastus kongosanensis* L.) mineral content and nutritional value. Iron **(A)**, magnesium **(B)**, manganese **(C)**, potassium **(D)**, phosphorous **(E)**, and calcium **(F)** in bamboo species under 100 and 200 mg L^-1^ copper in four replications. Control, 0 mg L^-1^ Cu, Bs (β-sitosterol, 100 mg L^-1^), Eb (eucalyptus biochar, %10). The lowercase letters (a, b, c, etc.) demonstrated significant differences among the treatments (Duncan, *p <* 0.05). The black dots show the outliers.

The presence of Cu reduces plant nutrient availability. However, integrating Bs and Eb substantially improved the mineral content in bamboo species. We found a significant difference between each of the treatments in terms of elevating nutrient value (p<0.001) (**Figure 2**). The treatments, including the combination of Bs+Eb, Eb alone, and Bs alone, exhibited the most substantial enhancement in mineral content. Specifically, there was a 35% rise in Fe, 44% in Mg, 25% in Mn, 12% in K, 20% in P, and 23% in Ca. Similarly, there was a 27% increase in Fe, a 32% increase in Mg, a 19% increase in Mn, a 9% increase in K, a 15% increase in P, and an 18% increase in Ca with the Eb treatment. Lastly, the Bs treatment resulted in an 18% increase in Fe, a 25% increase in Mg, a 12% increase in Mn, a 7% increase in K, a 10% increase in P, and a 10% increase in Ca. Nevertheless, adding Bs and Eb, both individually and in combination, considerably improved the nutritional value of plants under Cu concentrations of 100 and 200 mg L^-1^. The most significant increase was observed in the presence of Bs+Eb at concentrations of 100 and 200 mg L^-1^, with a 178% rise in Bs +Eb, a 157% rise in Fe, a 120% rise in Mg, a 190% rise in Mn, an 87% rise in K, a 90% rise in P, and a 99% rise in Ca, compared to respective controls. The exposure of 100 and 200 mg L^-1^ Cu results in a 51% reduction in plant nutritional value, a 64% reduction in Fe, a 48% reduction in Mg, a 68% reduction in Mn, a 58% reduction in K, a 72% reduction in P, and a 52% reduction in Ca.

### 3.3 The biochemical assessments and electrolyte leakage (H_2_O_2_, O_2_, MDA, EL)

**Figure 3:**
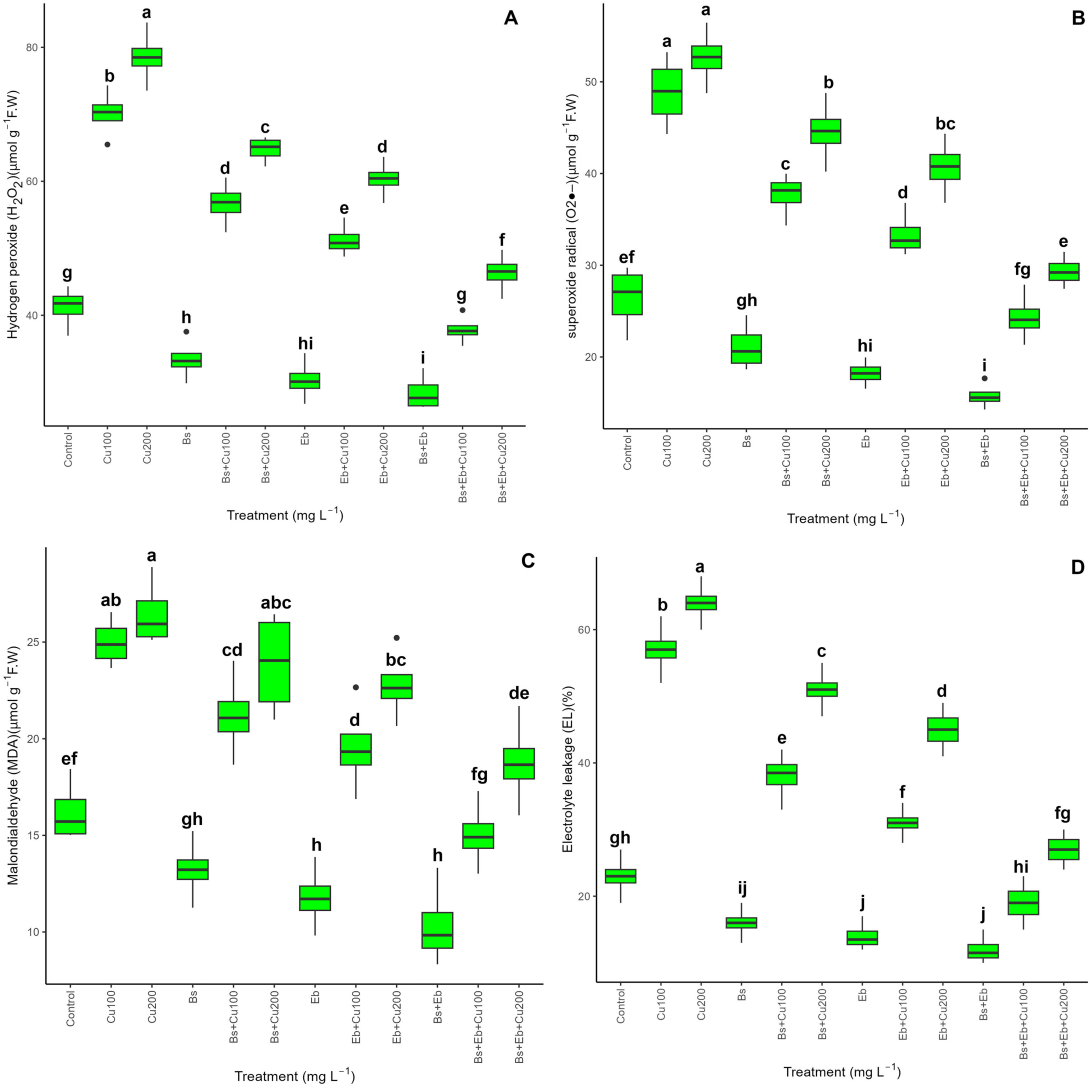
The impact of β-sitosterol and eucalyptus biochar on biochemical assessments and electrolyte leakage of bamboo (*Pleioblastus kongosanensis* L.). H_2_O_2_ **(A)**, O_2_ **(B)**, MDA **(C)**, and EL **(D)** in bamboo species under 100 and 200 mg L^-1^ copper in four replications. Control, 0 mg L^-1^ Cu, Bs (β-sitosterol, 100 mg L^-1^), Eb (eucalyptus biochar, %10). The lowercase letters (a, b, c, etc.) demonstrated significant differences among the treatments (Duncan, *p <* 0.05). The black dots show the outliers.

Considering electrolyte leakage and biochemical assessment, a significant disparity was observed between the treatments and the control groups (p<0.001). Based on **Figure 3**, adding both Bs+Eb, individually or in combination, decreased H_2_O_2_, O_2_^-.^, MDA, and EL levels without Cu exposure. However, the treatments with Bs+Eb show the most significant decrease in oxidative stress and cell injury. Conversely, adding Bs and Eb drastically lowered biochemical integrity and electrolytes in plants exposed to 100 and 200 mg L^-1^ Cu. The treatments Bs+Eb+100 mg L^-1^ Cu and Bs+Eb+200 mg L^-1^ Cu exhibited the most substantial drops in H_2_O_2_, O_2_^-.^, MDA, and EL, with an overall reduction of 41%. Specifically, there was a reduction of 41% in H_2_O_2_, 50% in O_2_^-.^, 39% in MDA, and 66% in EL compared to the control groups. Similarly, the treatments Bs+Eb+200 mg L^-1^ Cu showed a 44% reduction in O_2_^-.^, 29% in MDA, and 57% in EL. The single form of Eb worked more effectively in decreasing oxidative stress induced by Cu than Bs.

### 3.4 Plant osmolyte (soluble carbohydrate and proline) and glycine betaine (GB) content

**Figure 4:**
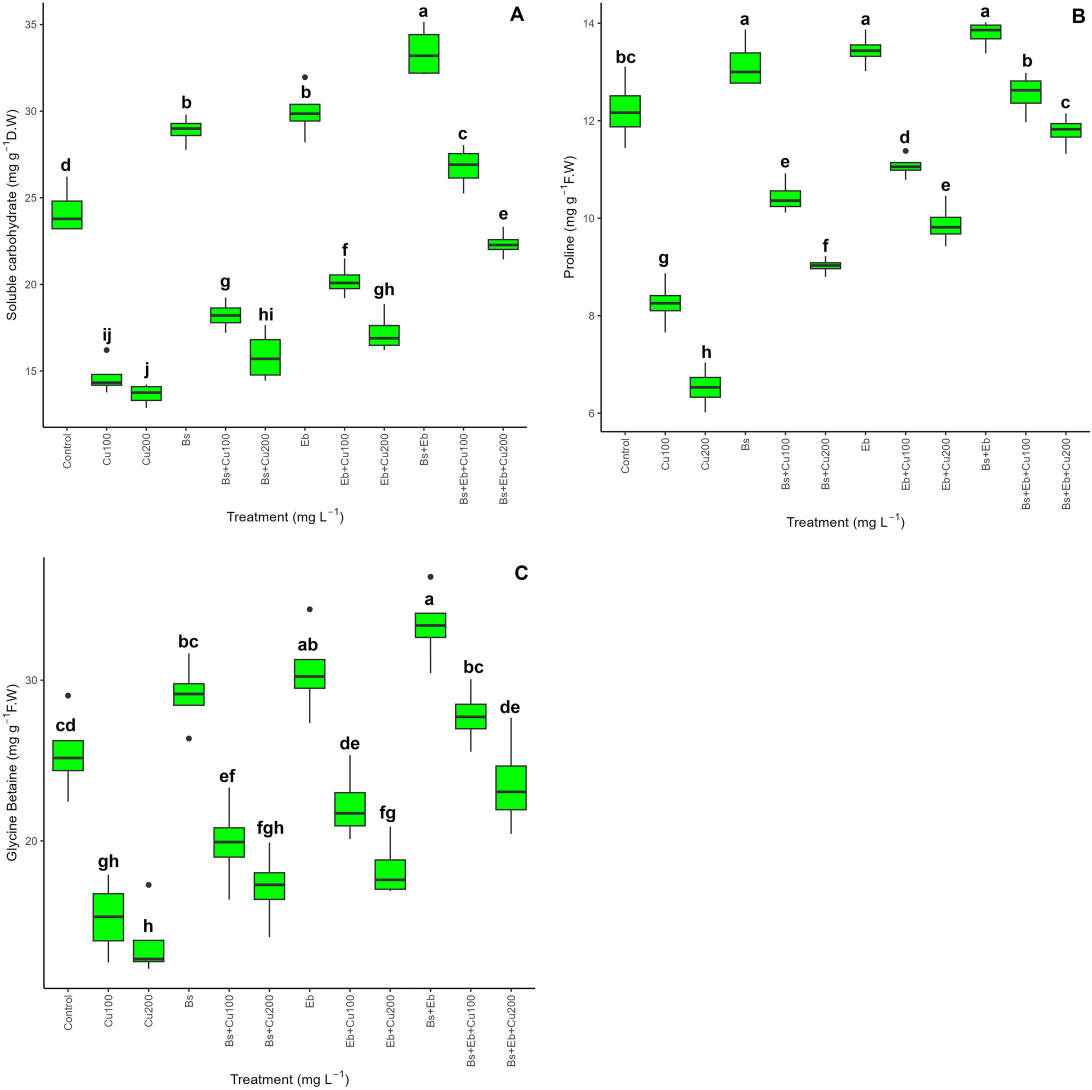
The impact of β-sitosterol and eucalyptus biochar on bamboo (*Pleioblastus kongosanensis* L.) osmolyte and glycine betaine (GB) content. Soluble carbohydrate content **(A)**, proline content **(B)**, and GB content **(C)** in bamboo species under 100 and 200 mg L^-1^ copper in four replications. Control, 0 mg L^-1^ Cu, Bs (β-sitosterol, 100 mg L^-1^), Eb (eucalyptus biochar, %10). The lowercase letters (a, b, c, etc.) demonstrated significant differences among the treatments (Duncan, *p <* 0.05). The black dots show the outliers.

A combination of Bs and Eb had a beneficial effect on enhancing the levels of plant osmolytes, specifically proline and GB, in both Cu-treated and non-treated plants. The data shows a substantial variation in the soluble carbohydrates, proline, and GB content levels when Bs and Eb were included in the treatments (p > 0.001). In plants not affected by Cu toxicity, the most prominent upgrades were observed using the combination of Bs+Eb, Eb, and Bs. These resulted in a 37%, 23%, and 19% increase in soluble carbohydrates, a 12%, 10%, and 7% increase in proline, and a 31%, 20%, and 14% increase in GB content. On the other hand, the most substantial increases in plants exposed to Cu were observed when plants were treated with Bs+EB under 100 and 200 mg L^-1^ Cu concentrations. These treatments resulted in an 82% and 63% increase in soluble carbohydrates, a 52% and 80% increase in proline content, and an 82% and 72% increase in GB content compared to the controls (**Figure 4**).

### 3.5 Antioxidant enzyme activity

**Figure 5:**
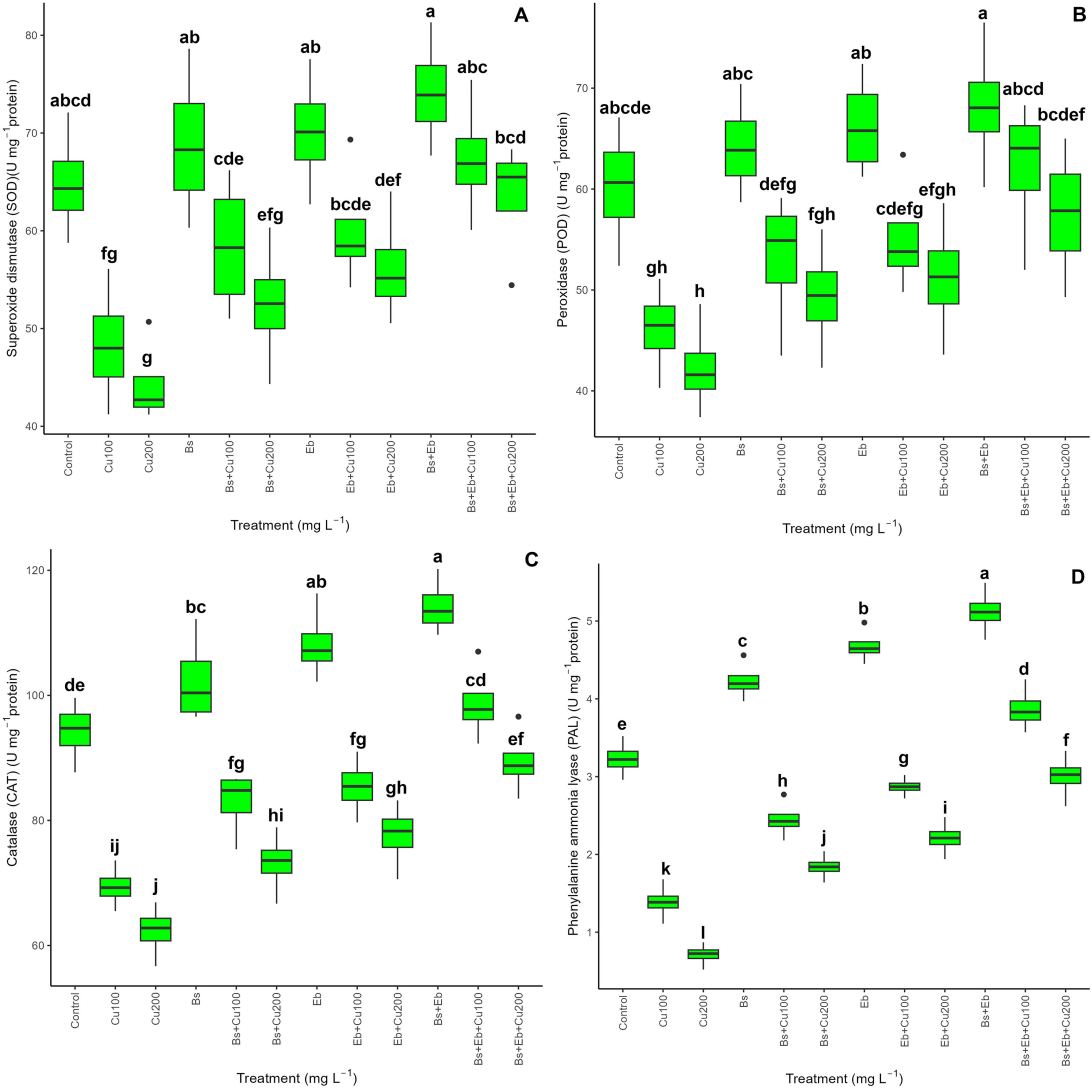
The impact of β-sitosterol and eucalyptus biochar on antioxidant enzyme activities of bamboo (*Pleioblastus kongosanensis* L.). Superoxide dismutase **(A)**, peroxidase **(B)**, catalase **(C)**, and phenylalanine ammonia-lyase **(D)** in bamboo species under 100 and 200 mg L^-1^ copper in four replications. Control, 0 mg L^-1^ Cu, Bs (β-sitosterol, 100 mg L^-1^), Eb (eucalyptus biochar, %10). The lowercase letters (a, b, c, etc.) demonstrated significant differences among the treatments (Duncan, *p <* 0.05). The black dots show the outliers.

The data on antioxidants revealed a significant difference in SOD, POD, CAT, and PAL activity between the treatments (P<0.001). Both the single and combined forms of Bs and Eb significantly elevated overall antioxidant activity. Including Bs and Eb resulted in a higher level among all antioxidant enzymes, irrespective of Cu levels. The most dramatic boost in antioxidant enzyme activity was observed when bringing together Bs and Eb with 100 and 200 mg L^-1^ Cu. This resulted in a 39% and 43% rise in SOD activity, a 34% and 36% rise in POD activity, a 42% and 43% rise in CAT activity, and a 178% and 222% rise in PAL activity compared to respective controls. In addition, the results revealed that the individual forms of Bs+100 mg L^-1^, Bs+200 mg L^-1^, Eb+100 mg L^-1^, and Eb+200 mg L^-1^ contributed to higher levels of antioxidant enzyme activity. We found a 21% increment in SOD, a 15% in POD, a 19% in CAT, and a 76% in PAL activity for Bs+100 mg L^-1^ Cu. As for Bs+200 mg L^-1^ Cu, there was an 18% increase in SOD, 16% in POD, 17% in CAT, and 159% in PAL activity. Similarly, Eb+100 mg L^-1^ showed a 24% increase in SOD, a 19% increase in POD, a 23% increase in CAT, and a 106% increase in PAL. Lastly, Eb+200 mg L^-1^ Cu treatment exhibited a 26% increase in SOD, a 21% increase in POD, a 24% increase in CAT, and a 211% increase in PAL activity in comparison with their control treatments (**Figure 5**).

### 3.6 Glyoxalase cycle activity and methylglyoxal content

**Figure 6:**
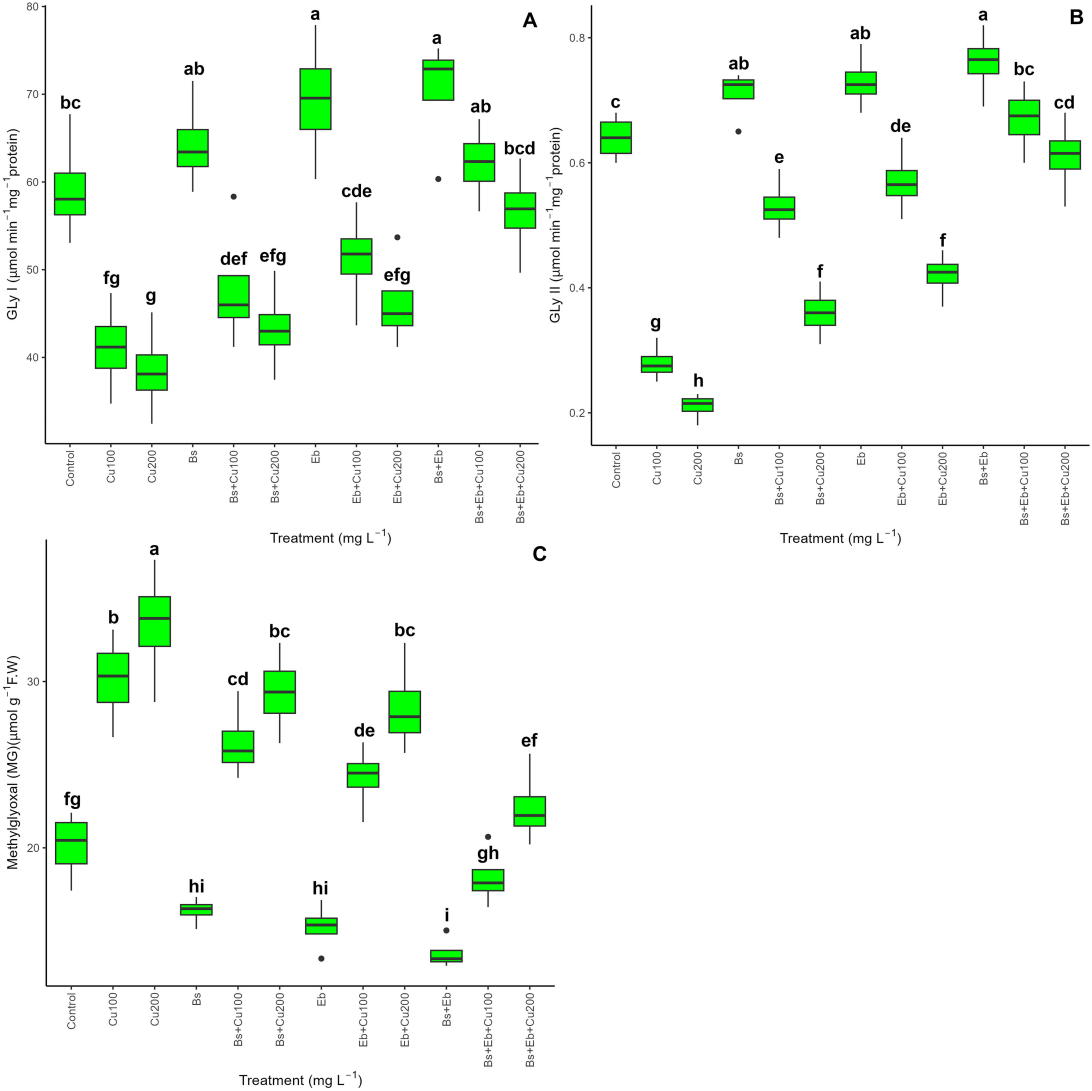
The impact of β-sitosterol and eucalyptus biochar on glyoxalase cycle activity and bamboo’s methylglyoxal (MG) content (*Pleioblastus kongosanensis* L.). Gly I **(A)**, Gly II **(B)**, and MG content **(C)** in bamboo species under 100 and 200 mg L^-1^ copper in four replications. Control, 0 mg L^-1^ Cu, Bs (β-sitosterol, 100 mg L^-1^), Eb (eucalyptus biochar, %10). The lowercase letters (a, b, c, etc.) demonstrated significant differences among the treatments (Duncan, *p <* 0.05). The black dots show the outliers.

The results demonstrated that whereas glyoxalase enzymes (Gly I and Gly II) were dramatically reduced by 100 mg L^-1^ and 200 mg L^-1^ Cu, Gly I and Gly II were increased in bamboo plants with and without Cu exposure (p<0.001) upon the addition of 80 mg L^-1^ Bs and 80 mg L^-1^ Eb, both singularly and in combination. Compared to controls, the combination of Bs+Eb+100 mg L^-1^ Cu and Bs+Eb+200 mg L^-1^ Cu resulted in the highest increase in Gly I and Gly II, with increases in Gly I of 51% and 47% and Gly II of 139% and 190%. Furthermore, adding Bs and Eb dramatically decreased the levels of MG in Cu-exposed bamboo (P <0.001). This highlights the possible function of the glyoxalase enzyme system in plants’ core defensive mechanism. Furthermore, the presence of both Bs and Eb, either individually or together, resulted in a reduction in MG content. Specifically, combining Bs with 100mg L^-1^ Cu resulted in a 12% reduction in MG content. Similarly, combining Bs with 200mg L^-1^ Cu led to a 12% drop. On the other hand, combining Eb with 100mg L^-1^ Cu resulted in a 19% decrease, while combining Eb with 200mg L^-1^ Cu led to a 14% decrease (**Figure 6**).

### 3.7 Non-enzymatic antioxidants (reduced glutathione, oxidized glutathione, and ascorbic acid)

**Figure 7:**
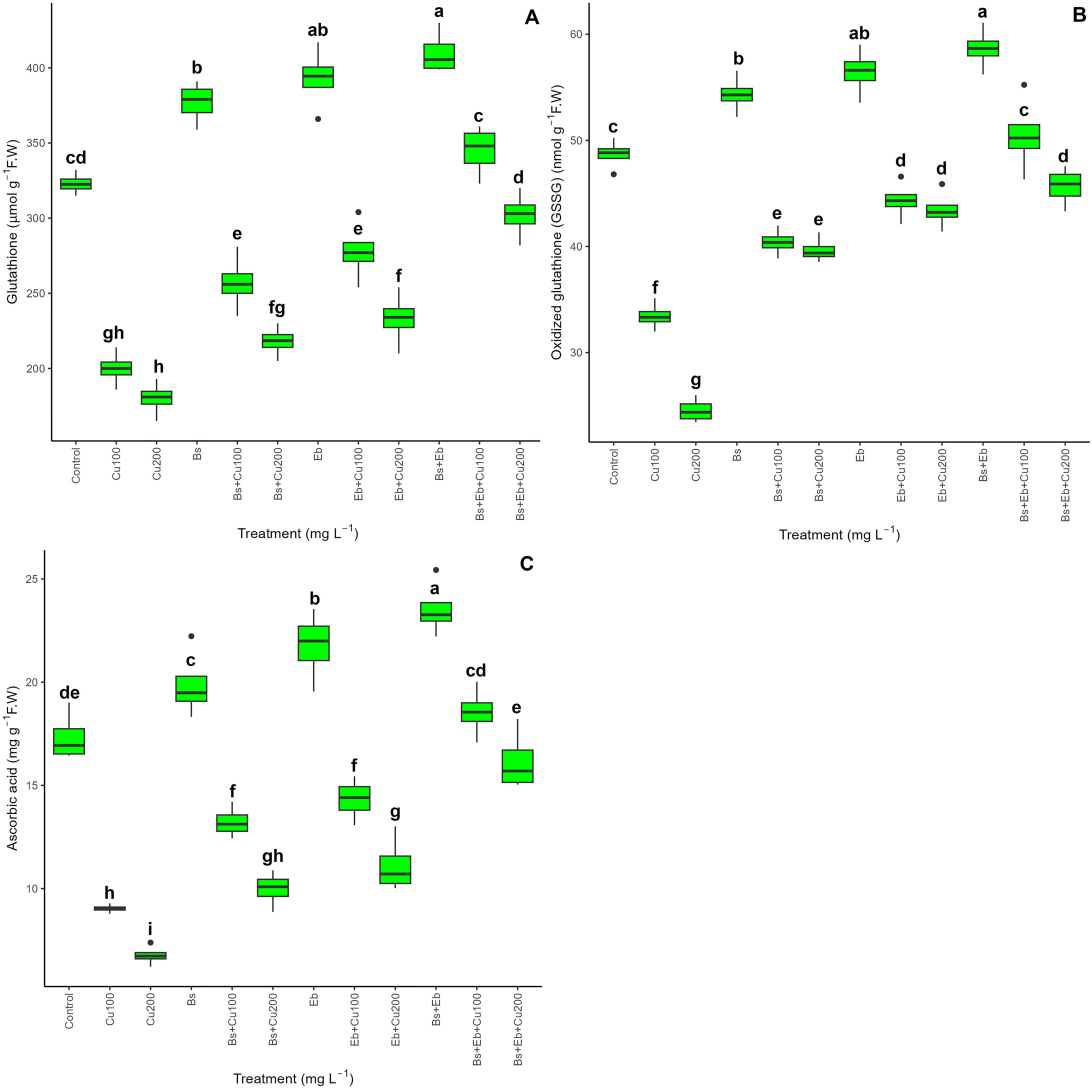
The impact of β-sitosterol and eucalyptus biochar on non-enzymatic activities of bamboo (*Pleioblastus kongosanensis* L.). Reduced glutathione **(A)**, oxidized glutathione **(B)**, and ascorbic acid **(C)** in bamboo species under 100 and 200 mg L^-1^ copper in four replications. Control, 0 mg L^-1^ Cu, Bs (β-sitosterol, 100 mg L^-1^), Eb (eucalyptus biochar, %10). The lowercase letters (a, b, c, etc.) demonstrated significant differences among the treatments (Duncan, *p <* 0.05). The black dots show the outliers.

The data demonstrated an integral disparity in non-enzymatic antioxidant activity across the treatments, as shown by reduced glutathione (GSH) levels, oxidized glutathione (GSSG), and ascorbic acid (AsA) (P<0.001). Bs and Eb increased non-enzymatic antioxidant activities with and without Cu concentrations. Both Bs and Eb, either alone or in combination, enhanced non-enzymatic activities in normal plants (without Cu), resulting in higher levels of GSH, GSSG, and AsA. The combination of Bs + Eb +100 mg L^-1^ Cu and Bs + Eb +200 mg L^-1^ Cu led to the most substantial increases, with a 72% and 67% rise in GSH, 51% and 86% increase in GSSG, and 105% and 139% increase in AsA in bamboo plants compared to their control treatments. Additionally, the addition of Bs (Bs+100 and Bs+200) and Eb (Eb+100 mg L^-1^ and Eb+200 mg L^-1)^ individually also raised non-enzymatic antioxidants by 28%, 21%, 39%, and 29% for GSH; 20%, 61%, 32%, and 77% for GSSG; and 46%, 47%, 58%, and 64% for AsA compared to the controls, respectively (**Figure 7**).

### 3.8 Plant water indexes: relative water content, water content, and the leaf water potential

**Figure 8:**
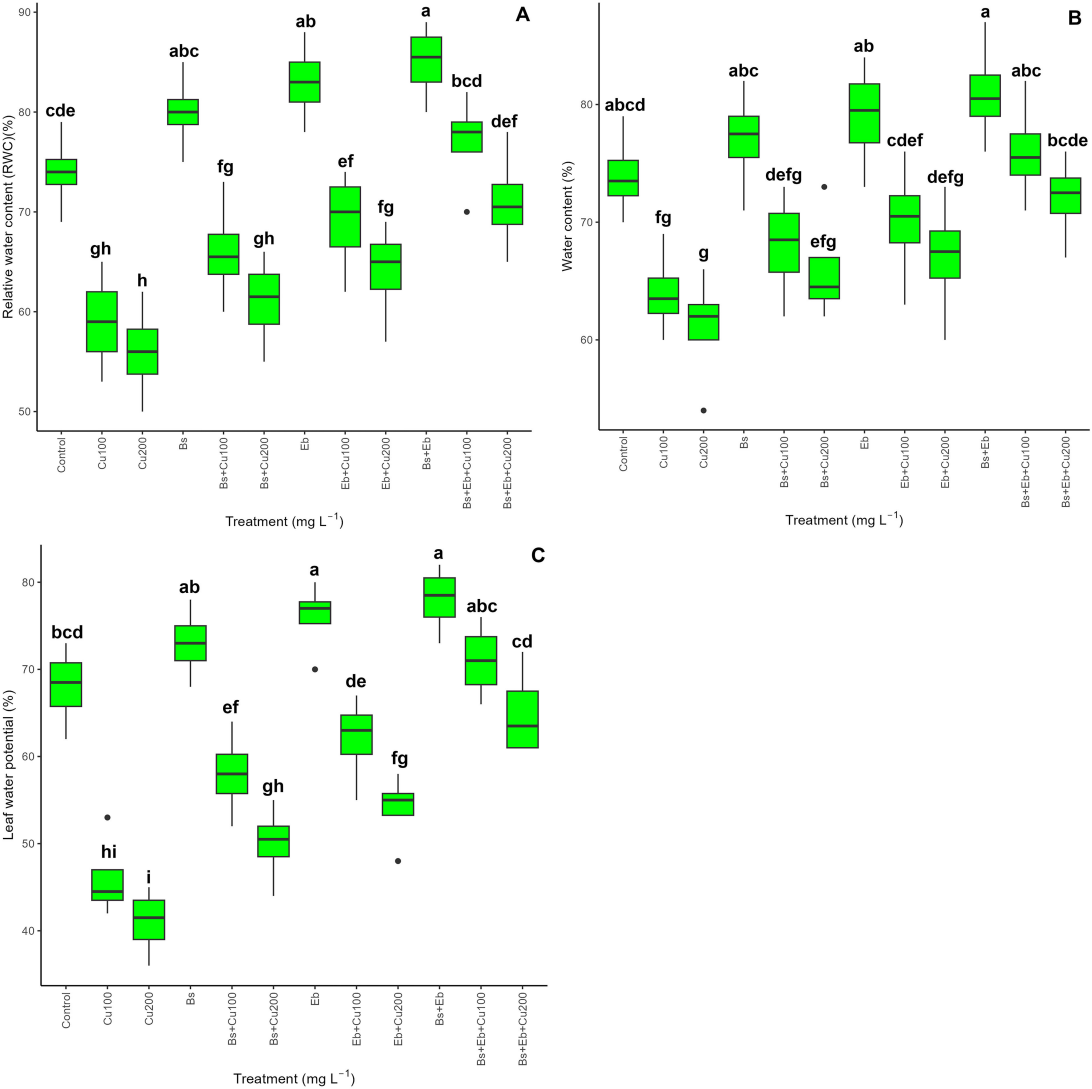
The impact of β-sitosterol and eucalyptus biochar on bamboo (*Pleioblastus kongosanensis* L.) plant water content. Relative water content **(A)**, water content **(B)**, and leaf water potential **(C)** in bamboo species under 100 and 200 mg L^-1^ copper in four replications. Control, 0 mg L^-1^ Cu, Bs (β-sitosterol, 100 mg L^-1^), Eb (eucalyptus biochar, %10). The lowercase letters (a, b, c, etc.) demonstrated significant differences among treatments (Duncan, *p <* 0.05). The black dots show the outliers.

Copper (100 and 200 mg L^-1^) reduced relative water content (RWC) (20% and 24%), water content (13%, 17%), and the leaf water potential (32% and 39%) in compared with control groups (0 mg L^-1^ Cu) (p<0.001) as expected. However, Bs and Eb, in both single and combo forms, increased water content indexes significantly (p<0.001), which showed that adding Bs and Eb positively impacts increasing plant water content under Cu stress. The greatest increase in plant water content was in the group where Bs and Eb were applied together, which was highlighted by a 30% and 26% increase in RWC, 18% and 18% in water content, and a 54% and 58% increase in leaf water potential revealing Bs and Eb might have a strong ability to increase plant water indexes (**Figure 8**).

### 3.9 Impact of β-sitosterol and eucalyptus biochar on chlorophyll pigments, carotenoid levels, and gas exchange parameters in bamboo plants under Cu levels

**Figure 9:**
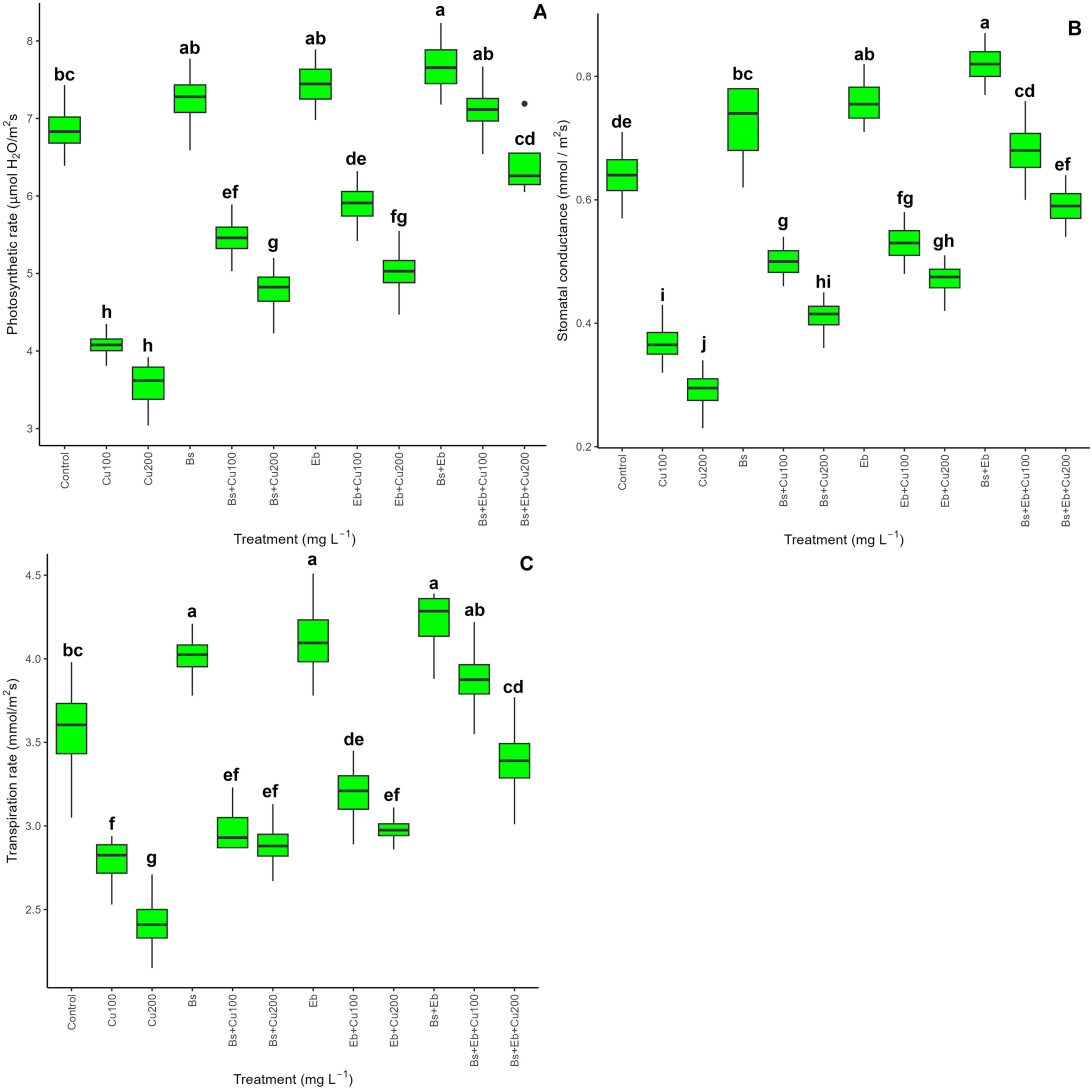
The impact of β-sitosterol and eucalyptus biochar on bamboo (*Pleioblastus kongosanensis* L.) gas exchange parameters. Photosynthetic rate **(A)**, stomatal conductance **(B)**, and transpiration rate **(C)** in bamboo species under 100 and 200 mg L^-1^ Cu in four replications. Control, 0 mg L^-1^ Cu, Bs (β-sitosterol, 100 mg L^-1^), Eb (eucalyptus biochar, %10). The lowercase letters (a, b, c, etc.) demonstrated significant differences among the treatments (Duncan, p < 0.05). The black dots show the outliers.

The results showed a significant difference (P<0.001) in the chlorophyll pigments and carotenoid levels in bamboo plants with and without Cu exposure. Both Bs and Eb, whether used alone or in combination, led to a significant increase in Chl a, Chl b, total chlorophyll, and carotenoid contents. The greatest increase in photosynthetic pigments was observed in the groups treated with Bs+Eb+100 mg L^-1^ and Bs+Eb + 200 mg L^-1^, showing a 74% and 88% increase in Chl a, a 46% and 68% increase in Chl b, a 54% and 77% increase in total chlorophyll, and an 82% and 154% increase in carotenoids compared to the controls, respectively (**Table 2**). Similarly, gas exchange indexes, including photosynthetic rate, stomatal conductance, and transpiration rate, also showed significant differences (P<0.001) across treatments with and without Cu exposure. The combination of Bs+Eb under 100 mg L^-1^ and 200 mg L^-1^ Cu resulted in the highest increase in gas exchange indexes, with a 74% and 81% rise in photosynthesis rate, an 83% and 103% rise in stomatal conductance, and a 39% and 40% rise in transpiration rate compared to their respective controls (**Figure 9**). These findings suggest that both Bs and Eb, when used individually, have the potential to significantly increase photosynthetic pigments and gas exchange parameters in bamboo plants under different Cu concentrations (100 and 200 mg/L^-1^).

**Table 2:** The effect of β-sitosterol and eucalyptus biochar on chlorophyll pigments (Chlorophyll-a, Chlorophyll-b, Total Chlorophyll, and Carotenoid) in bamboo plants under Cu toxicity.

Treatment	Chlorophyll-a (mg g^-1^ F.w.)	Chlorophyll-b (mg g^-1^ F.w.)	Chlorophyll a+b (mg g^-1^ F.w.)	Caratenoids (mg g^-1^ F.w.)
control	6.03± 0.13^cd^	7.23 ± 0.14^bc^	13.26 ± 0.32^c^	2.88 ± 0.01^cd^
100 mg L^-1^ Cu	3.76 ± 0.13^h^	5.21 ± 0.14^g^	9.22 ± 0.32^h^	1.67 ± 0.01^i^
200 mg L^-1^ Cu	3.12 ± 0.13^i^	4.16 ± 0.14^h^	7.28 ± 0.32^i^	1.09 ± 0.01^j^
100 mg L^-1^ Bs	6.98± 0.13^ab^	7.89 ± 0.14^a^	14.87 ± 0.32^ab^	2.23 ± 0.01^fg^
100 mg L^-1^ Bs + 100 mg L^-1^ Cu	5.03± 0.13^ef^	6.43 ± 0.14^de^	11.46 ± 0.32^ef^	2.31 ± 0.01^f^
100 mg L^-1^ Bs + 200 mg L^-1^ Cu	4.23 ± 0.13^gh^	5.77 ± 0.14^f^	10 ± 0.32^gh^	1.98 ± 0.01^h^
10% Eb	7.12± 0.13^a^	8.05 ± 0.14^a^	15.17.± 0.32^a^	3.45± 0.01^b^
10% Eb + 100 mg L^-1^ Cu	5.32± 0.13^e^	6.76 ± 0.14^cd^	12.08 ± 0.32^de^	2.55 ± 0.01^e^
10% Eb + 200 mg L^-1^ Cu	4.67 ± 0.13^fg^	6.12 ± 0.14^ef^	10.79 ± 0.32^fg^	2.11 ± 0.01^gh^
100 mg L^-1^ Bs + 10% Eb	7.21± 0.13^a^	8.21 ± 0.14^a^	15.42 ± 0.32^a^	3.65 ± 0.01^a^
100 mg L^-1^ Bs +10% Eb + 100 mg L^-1^ Cu	6.55± 0.13^bc^	7.65 ± 0.14^ab^	14.2 ± 0.32^b^	3.04 ± 0.01^c^
100 mg L^-1^ Bs +10% Eb + 200 mg L^-1^ Cu	5.88± 0.13^d^	7.01 ± 0.14^c^	12.89 ± 0.32^cd^	2.77 ± 0.01^d^

The data indicated the mean ± standard error of four repetitions. The treatments included different levels of β-sitosterol and eucalyptus biochar individually or in combination under four concentrations of Cu. The lowercase letters (a, b, c, d, etc.) display significant differences among treatments analyzed by Duncan’s test (p< 0.05).

### 3.10 Plant biomass and plant growth

**Figure 10:**
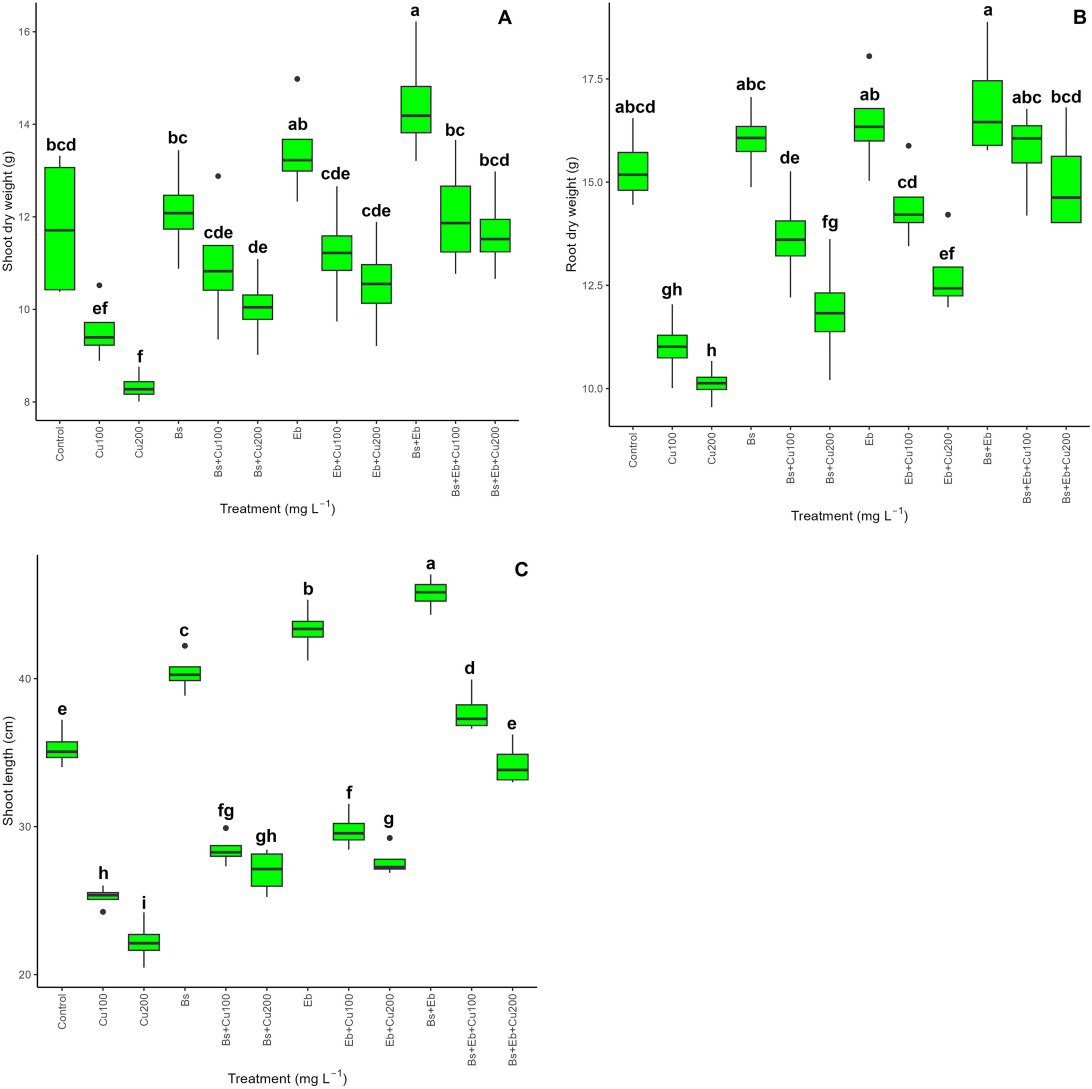
The impact of β-sitosterol and eucalyptus biochar on bamboo (*Pleioblastus kongosanensis* L.) plant biomass and plant growth indexes. Shoot dry weight **(A)**, root dry weight **(B)**, and shoot length **(C)** in bamboo species under 100 and 200 mg L^-1^ copper in four replications. Control, 0 mg L^-1^ Cu, Bs (β-sitosterol, 100 mg L^-1^), Eb (eucalyptus biochar, %10). The lowercase letters (a, b, c, etc.) demonstrated significant differences among the treatments (Duncan, p < 0.05). The black dots show the outliers.

The data indicated an apparent disparity in plant biomass and plant growth indexes across the treatments (P<0.001) (**Figure 10**). Bamboo plants exposed to doses of Cu of 100 mg L^-1^ and 200 mg L^-1^ experienced significant reductions in shoot dry weight (19% and 29% reduction), root dry weight (28% and 34% reduction), and shoot length (28% and 37% reductions). The combined administration of Bs and Eb, either individually (Bs+100 mg L^-1^ Cu), (Bs+200 mg L^-1^ Cu), (Eb+100 mg L^-1^ Cu), (Eb+200 mg L^-1^ Cu), or in combination (Bs+Eb+100 mg L^-1^ Cu), (Bs+Eb+200 mg L^-1^ Cu), resulted in significant modifications in plant biomass and plant growth indices. These benefits were observed as a 15%, 20%, 17%, 26%, 26%, and 40% raise in shoot dry weight, a 24%, 17%, 31%, 26%, 43%, and 48% rise in root dry weight, and a 12%, 21%, 18%, 24%, 49%, and 54% rise in shoot length, respectively. Conversely, as estimated, the treatments lacking different Cu concentrations exhibited a growing tendency in plants’ shoot and root dry weight and shoot length when Bs and Eb were added, individually or in combination (**Figure 10**) (**Table 3**).

**Table 3:** The effect of β-sitosterol and eucalyptus biochar alone and in combination with different Cu levels on bamboo plants *Pleioblastus kongosanensis* L. SHDB and RDB, relative to control.

Treatments	SHDB (%)	RDB (%)	SHL(cm)
100 mg L^-1^ Cu	19% ↓	28% ↓	28% ↓
200 mg L-1 Cu	29% ↓	34% ↓	37% ↓
100 mg L-1 Bs	3% ↑	4% ↑	14% ↑
100 mg L-1 Bs + 100 mg L-1 Cu	7% ↓	10% ↓	19% ↓
100 mg L-1 Bs + 200 mg L-1 Cu	14% ↓	22% ↓	23% ↓
10% Eb	14% ↑	7% ↑	22% ↑
10% Eb + 100 mg L-1 Cu	4% ↓	5% ↓	15% ↓
10% Eb + 200 mg L-1 Cu	10% ↓	16% ↓	21% ↓
100 mg L-1 Bs + 10% Eb	22% ↑	10% ↑	29% ↑
100 mg L-1 Bs +10% Eb + 100 mg L-1 Cu	2% ↑	3% ↑	7% ↑
100 mg L-1 Bs +10% Eb + 200 mg L-1 Cu	1% ↓	2% ↓	3% ↓

↓ indicates decrease and ↑ indicates increase.

### 3.11 Translocation factor, bioaccumulation factor, and tolerance index

The analysis of data obtained by calculating the bioaccumulation factor (BAF) of bamboo plant organs (roots and shoots) revealed a significant difference in BAF among treatments under different Cu doses with the addition of Bs and EB (P<0.001). The treatments combining Bs + Eb with 100 mg L^-1^ Cu and 200 mg L^-1^ Cu exhibited the most substantial reduction in BAF, with a 51% and 45% decrease in shoots and a 33% and 38% reduction in roots, respectively, compared to their controls. Additionally, Bs and Eb in individual forms (Bs+100 mg L^-1^ Cu), (Bs+200 mg L^-1^ Cu), (Eb+100 mg L^-1^ Cu), and (Eb+200 mg L^-1^ Cu) also significantly reduced BAF in bamboo shoots and roots by 19%, 17%, 29%, and 22% in shoots, and 18%, 18%, 23%, and 24% in roots compared to respective controls.

The translocation factor (TF) exhibited a difference among treatments in the leaves (P<0.001), with Bs and Eb in both individual and combination forms resulting in reduced TF. The most substantial reduction was observed in the group of Bs+Eb under 100 mg L^-1^ Cu and 200 mgL^-1^ Cu, which showed a 27% and 10% drop in TF compared to their controls.

The shoot and root tolerance index (TI) also displayed significant differences across treatments (p<0.001). Incorporating Bs and Eb, either individually or in combination, significantly increased TI in both the shoots and roots. Notably, the combination of Bs+Eb demonstrated the highest TI under 100 mg L^-1^ Cu and 200 mg L^-1^ Cu, with a 26% and 40% increase in bamboo shoots and a 42% and 48% increase in bamboo roots compared with control treatments (**Table 4**).

**Table 4:** Changes in the bioaccumulation factor (BAF), and tolerance index (TI) of shoot and roots upon β-sitosterol and eucalyptus biochar in individual or in combination form under 100, 150, and 200 mg L^-1^ Cu.

Treatments	TF (leaf)	BAF(leaf)	BAF(root)	TI (root)	TI (shoot)
control	0	0	0	1.00 ± 0.03^abc^	1.00 ± 0.03^abcd^
100 mg L^-1^ Cu	0.412 ± 0.002^ab^	0.19 ± 3.03^a^	0.463 ± 0.001^a^	0.72 ± 0.03^ef^	0.824 ± 0.03^cd^
200 mg L^-1^ Cu	0.42 ± 0.002^ab^	0.111 ± 3.03^d^	0.263 ± 0.001^e^	0.66 ± 0.03^f^	0.718± 0.03^d^
100 mg L^-1^ Bs	0	0	0	1.049 ± 0.03^abc^	1.04 ± 0.03^abc^
100 mg L^-1^ Bs + 100 mg L^-1^ Cu	0.407 ± 0.002^ab^	0.154 ± 3.03^b^	0.378 ± 0.001^b^	0.89± 0.03^bcde^	0.95± 0.03^abcd^
100 mg L^-1^ Bs + 200 mg L^-1^ Cu	0.423 ± 0.002^ab^	0.19 ± 3.03^a^	0.215 ± 0.001^f^	0.77 ± 0.03^def^	0.87 ± 0.03^bcd^
10% Eb	0	0	0	1.076 ± 0.01ab	1.16 ± 0.03^ab^
10% Eb + 100 mg L^-1^ Cu	0.384 ± 0.002^c^	0.135 ± 3.03^c^	0.354 ± 0.001^c^	0.94 ± 0.03^abcd^	0.97 ± 0.03^abcd^
10% Eb + 200 mg L^-1^ Cu	0.43 ± 0.002^a^	0.09 ± 3.03^e^	0.199 ± 0.001^g^	0.88± 0.03^cde^	0.915± 0.03^bcd^
100 mg L^-1^ Bs + 10% Eb	0	0	0	1.106 ± 0.01^a^	1.24± 0.03^a^
100 mg L^-1^ Bs +10% Eb + 100 mg L^-1^ Cu	0.301 ± 0.002^d^	0.093 ± 3.03^e^	0.310 ± 0.001^d^	1.02 ± 0.03^abc^	1.039 ± 0.03^abcd^
100 mg L^-1^ Bs +10% Eb + 200 mg L^-1^ Cu	0.379 ± 0.002^c^	0.06 ± 3.03^f^	0.161 ± 0.001^h^	0.98 ± 0.03^abc^	1.01 ± 0.03^abcd^

The data display the mean ± standard error of four replicates. Lowercase letters show differences among groups (Duncan p < 0.001).

## 4 Discussion

While previous studies have primarily focused on the general trend of using forms of biochar compounds as single external additives, this study contributes to the field by combining β-sitosterol (Bs) and eucalyptus biochar (Eb). In this context, we discussed the impact of this combination in terms of application area, theoretical contribution, or possible ecological implications.

In our experiments, the rise in Cu stress remarkably triggered the accumulation of ROS compounds, including hydrogen peroxide (H_2_O_2_) and superoxide (O_2_•−), as well as led to damage to plant membranes with higher rates of lipoperoxidation (MDA) and EL in bamboo. Many studies on different plant species under Cu toxicity, such as *Artemisia annua* (Zehra et al., 2020) and *Arabidopsis* (Pető et al., 2013; Wang et al., 2022; Maksymiec et al., 2007) reported corroborating results. The data showed that while the concentrations of Cu reduce antioxidant (SOD, CAT, POD, and PAL) activity and non-antioxidants (GSH, GSSG, and AsA) in bamboo, integrating Bs in single and combined forms with Eb (10%) increased antioxidant and non-antioxidant indexes of plants. This might be related to improved redox status by β-sitosterol via activation of the antioxidant signal pathway (Xie et al., 2022). Because β-sitosterol, as a modest antioxidant in lipid media, can ideally reduce oxidative stress (Xie et al., 2022). This increasing antioxidant activity and non-antioxidant content by biochar addition were reported in several works (Sharma et al., 2012; Emamverdian and Ding, 2017b; Mansoor et al., 2022; Emamverdian et al., 2024; Alharbi et al., 2023; Mishra et al., 2019; Shahzad et al., 2021). In parallel studies, Kosolsaksakul et al. (2018); Alharbi et al. (2023), and Zheng et al. (2017) have all reported the protective role of biochar and Bs against oxidative stress caused by HMs.

The process of stimulating the glyoxalase cycle (Gly I and Gly II), which is a significant stress marker, includes two steps: conversion of MG to SLG via GSH in the presence of Gly I and then production of D-lactic acid from SLG by Gly II action (Hasanuzzaman et al., 2018). Applying Bs and Eb remarkably reduced MG content in the study with more glyoxalase (Gly I and Gly II) activity. The impact of biochar on increasing glyoxalase enzymes and the reduction of MG were also reported by Hasanuzzaman et al. (2021), which is in line with our data. In addition, Bs and Eb have significantly reduced H_2_O_2_, MDA, and EL content in *thymus* plants (Alharbi et al., 2023). This has also been reported in other studies (Huang et al., 2021; Elkeilsh et al., 2019), which can be shown as evidence overlapping our results in this study.

Furthermore, the results show that Bs and Eb significantly increase GB, proline, and soluble carbohydrates levels under Cu concentrations, which leads to bamboo plant osmoregulation, improves membrane stability, increases photosynthetic capacity, and reduces plant stress under Cu toxicity, which corroborates the results obtained by Alharbi et al. (2023) on *Thymus vulgaris*. Indeed, as a major compatible substance, GB plays a positive role in enhancing photosynthetic pigment intactness under stress (Emamverdian et al., 2022b) and is a protection barrier against photosynthetic components, such as O_2_-producing photosystem II and Rubisco (Altaf et al., 2020; Xu et al., 2018) here. Proline, on the other hand, might have functioned as a fixer for protein complexes in bamboo cells under Cu stress, a process that requires proteins to undergo conformational changes. Soluble carbohydrates might have been improved by stressed bamboo cells in providing energy and a carbon skeleton for sustaining cell development and organic molecule synthesis, which might have played an essential role in membrane turgidity and membrane stability in bamboo plants (Kishor et al., 2005; Arnao and Hernandez-Ruiz, 2019).

It was also reported that Bs influence pathways, including glutamic acid converting to 5-oxo proline and proline instead of going to alanine and pyruvate accumulation, preserving water plant content in stress conditions (Li et al., 2019). Our results show that working with Bs and Eb in single and combo forms remarkably increased bamboo relative water content and the leaf water potential, positively impacting plant photosynthetic efficiency. Specific structures of biochar, such as porous features, can preserve the plant water capacity and increase photosynthetic activity in plants under HM stress (Naeem et al., 2020; Sofy et al., 2021; Retamal-Salgado et al., 2017). Furthermore, data showed that adding Bs and Eb to bamboo plants under Cu doses increased nutrient content and reduced Cu uptake in bamboo roots, stems, and shoots. Here, Bs and Eb together might also have improved soil characteristics by promoting the uptake and absorbance of micronutrients and macronutrients such as Mg, Ca, P, K, Mn, and Fe (Retamal-Salgado et al., 2017), which converts soil structure into a high-quality agricultural fertilizer source and increase the nutrient status of targeted soil environment (Ge et al., 2019). Because nutrients are essential for plants that can reduce HM uptake, translocation, aggregation, and metal bioavailability, and they trap HMs by adsorbing on the substrate surface, chelating, or binding, as reported in maize by combined biochar and thiourea application in Cd-contaminated soil (Haider et al., 2021). On the other hand, some biochar types are also enriched and activated with cations, like K^+^, Mg^+2^, and Ca^+2^, for better adsorption capacity. These cation ions might have been present in plant tissue cell walls as amorphous buffers, potentially decreasing the absorption of Cu in bamboo species (Younis et al., 2016). In our study, Cu concentrations in plant organs such as root, stem, and shoot were reduced by using combined Bs and Eb, which limited Cu bioaccumulation and Cu translocation from soil to plants. The application of Eb possibly reduced Cu absorbance from the soil with the help of increasing soil pH through physical sorption, complication, precipitation, ion exchange, membrane filtration, and electrostatic interaction mechanisms, which in turn help reduce Cu availability in organs (Ali et al., 2013; Sfaxi-Bousbih et al., 2010). These changes were recently reported in Cd-stressed maize by Ahmad et al. (2018) and Haider et al. (2022).

The gene-level approaches showed that biochars can suppress the transcript expression of genes related to Cd transport and uptake and the movement of Cd to shoots through the apoplast, which limits Cd’s toxicity in plants (Abbas et al., 2017). In our study, while bamboo plants under Cu toxicity significantly reduced photosynthetic pigments and gas exchange parameters, applying Bs and Eb in both single and combined forms increased photosynthesis under 100 and 200 mg L^-1^ Cu concentrations. Combining sitosterol supplementation and biochar increased photosynthetic properties by improving enzymes taking place in the photosynthesis process was also reported previously (Maghsoudi et al., 2015). It was reported that the increase in photosynthesis rate in plants with the addition of Bs and Eb could be related to the reduction of hydroxyl radicals’ content, as indicated by previous studies on maize and rice (Abideen et al., 2020; Shahzad et al., 2021). However, this phenomenon should be supported by molecular studies showing gene expression data in relation to bamboo Cu-stress response/tolerance.

The bamboo plant growth obtained in this work under Cu stress might also have been improved by maintaining cell expansion and proliferation through maintaining water potential (Alharby and Fahad, 2020). In this context, Eb amendments in the soil putatively increased plant growth and production by boosting the soil’s physicochemical properties, such as pH, nutrient capacity, enzymatic activities, and water-holding capacity (Hassan et al., 2020). Maintaining water content capacity in the plant is an essential factor in increasing plant photosynthesis and biomass, as also reported by Zhou et al. (2017) and Abdelshafy Mohamad et al. (2020). The application of Bs was indeed reported to increase growth and biomass via improvement in the antioxidant system, such as the stimulation of the expression level of SOD and dehydrin genes in wheat (*Triticum aestivum*) (Elkeilsh et al., 2019). The findings of our study indicate that the presence of Cu in bamboo leads to a decrease in biomass. However, the introduction of Bs and Eb, either individually or in combination, resulted in an increase in both root and shoot dry weight, as well as shoot lengths. This could be due to the simultaneous power of Bs and Eb additives in increasing plant water content and antioxidant capacity, leading to increased photosynthesis, growth, and biomass.

The soil transformation resulting from biochar application enabled better photosynthetic function along with increased water content, which probably resulted from reduced toxic Cu ion availability and better stress alleviation (Hasnain et al., 2023; Yadav et al., 2023). In addition, the enhancement of beneficial microbial communities through biochar could promote nutrient cycling and the production of plant growth-promoting compounds (Hasnain et al., 2023; Yadav et al., 2023). This might explain the increased antioxidant capacity and activity of the glyoxalase cycle observed in the present work since microbial interaction often acts as a prime factor that modulates plant stress response. Moreover, the porous structure of biochar provides beneficial microorganisms with a habitable environment and thus supports plant health against unfavorable stress conditions (Bolan et al., 2023). Considering such indirect effects, our results are in agreement with the general view about the role of biochar in enhancing plant tolerance to HM stress, with a boosting effect of Bs as a plus. These findings are based on controlled lab conditions; therefore, field trials are needed to validate practical efficacy. While Bs+Eb improved Cu tolerance, molecular mechanisms require further investigation. Long-term soil Cu dynamics and plant-microbe interactions under Bs+Eb treatment also remain to be studied.

## 5 Conclusions

This study demonstrates that the combined application of β-sitosterol (Bs) and eucalyptus biochar (Eb) significantly enhances bamboo tolerance to copper (Cu) stress by improving redox homeostasis, nutrient uptake, and photosynthetic efficiency. The treatments reduced Cu accumulation in plant tissues while boosting antioxidant defenses, osmoregulation, and growth. These findings highlight the potential of Bs+Eb as a sustainable strategy for phytoremediation in Cu-contaminated soils. However, field validation and mechanistic studies are needed to assess long-term efficacy and scalability. Adsorption technologies and high-surface-area biochars have become crucial and cost-effective ways to mitigate metal pollution. In accordance with that, we examined bamboo responses to Cu toxicity at physiological and morphological levels under β-sitosterol (Bs), a cholesterol-like phytosterol, and the eucalyptus biochar (Eb) effect. According to our data, sustainable agriculture should include Bs and Eb treatments to restore heavy metal (HM)-contaminated soil bases. Because β-sitosterol and Eb, alone or in combination, significantly increased plant tolerance to Cu-induced stress by neutralizing ROS (oxidative stress), activating antioxidant and non-antioxidant defense systems, increasing glyoxalase enzymes activity, and improving osmoregulation thanks to higher proline and glycine betaine levels. Both compounds increased photosynthetic efficiency, gas exchange, and nutrient intake while reducing Cu transport and accumulation in plant structures. Their synergistic efficiency improved plant growth, biomass production, and water retention, reducing Cu toxicity. These results highlight the ability of Bs and Eb treatments to improve bamboo plant tolerance exposed to Cu stress, suggesting their practical use in sustainable farming and rehabilitating polluted environments. However, additional evaluation of Eb-Bs-induced indirect impact on the study results would be necessary. For example, Bs mediated with Eb treatment may initiate the expression of genes responsible for biomolecule synthesis components, such as PCs and MTs associated with Cu detoxification. Although much has been addressed, the detailed molecular mechanisms of how Eb, in conjunction with Bs treatment, modulates the plant molecular responses opposing stress while up-regulating genes involved in Cu detoxification and activation of stress signal transduction pathways have not been well addressed. Such a study investigating how the gene expression of antioxidant enzymes like SOD and CAT, metal transporters like HMAs, and stress-responsive transcription factors like SPL7 are affected by Eb+Bs would improve the understanding of stress tolerance mechanisms. Therefore, further research into these complex interactions will help explain the mechanisms by which Eb+Bs exert their impact on plant molecular physiology response in detail. Indeed, plant species’ scalability in polluted locations requires more study. Field operational trials must test multiple plant species for treatment, deploy remediation methods, and examine economic, social, and climatic issues to enhance this technology. While these findings demonstrate effective Cu stress mitigation in bamboo, the observed mechanisms, particularly the Bs-Eb synergy, warrant investigation in other agriculturally important species to assess broader applicability. Furthermore, while our results are specific to bamboo physiology, the observed redox regulation and nutrient management strategies may also provide a template for developing analogous approaches in other metal-stressed crops, subject to further testing. This research’s practical solution to HM contamination and food safety and eco-balance lays the groundwork for environmental restoration and sustainable agricultural methods. Addressing these areas further would broaden the approach’s role in soil health, crop production, and food security.

## Data Availability

The raw data supporting the conclusions of this article will be made available by the authors, without undue reservation.
