# Supplementary material for: Dual application of β-sitosterol and biochar reduces copper toxicity in bamboo via improved redox homeostasis
Source: Front Plant Sci. 2025 Aug 19;16:1554519. doi: 10.3389/fpls.2025.1554519 (PMC12403999; doi:10.3389/fpls.2025.1554519)
Supplement: Supplementary file 1 [file Table1.docx]

To ascertain the concentration of eucalyptus biochar, we referenced our prior research on the mitigation of heavy metals utilizing bamboo biochar. The concentration of eucalyptus biochar was established based on the concentration of bamboo biochar from our prior work:

Emamverdian, A., Ghorbani, A., Pehlivan, N., Li, Y., Zargar, M. and Liu, G., 2024. Bamboo biochar helps minimize Brassica phytotoxicity driven by toxic metals in naturally polluted soils of four mine zones. Environmental Technology & Innovation, 36, p.103753.

Concentrations of β-sitosterol were selected by other researchers on Thymus vulgaris, which we evaluated on bamboo species, too.

Alharbi K, Khan AA, Sakit Alhaithloul HA, Al-Harbi NA, Al-Qahtani SM, Aloufi SS, Abdulmajeed AM, Muneer MA, Alghanem SMS, Zia-Ur-Rehman M, Usman M, Soliman MH. Synergistic effect of β-sitosterol and biochar application for improving plant growth of Thymus vulgaris under heat stress. Chemosphere. 2023 Nov;340:139832. doi: 10.1016/j.chemosphere.2023.139832. Epub 2023 Aug 15. PMID: 37591372.
