# Supplementary figures and images for: Dual application of β-sitosterol and biochar reduces copper toxicity in bamboo via improved redox homeostasis
Source: Front Plant Sci. 2025 Aug 19;16:1554519. doi: 10.3389/fpls.2025.1554519 (PMC12403999; doi:10.3389/fpls.2025.1554519)

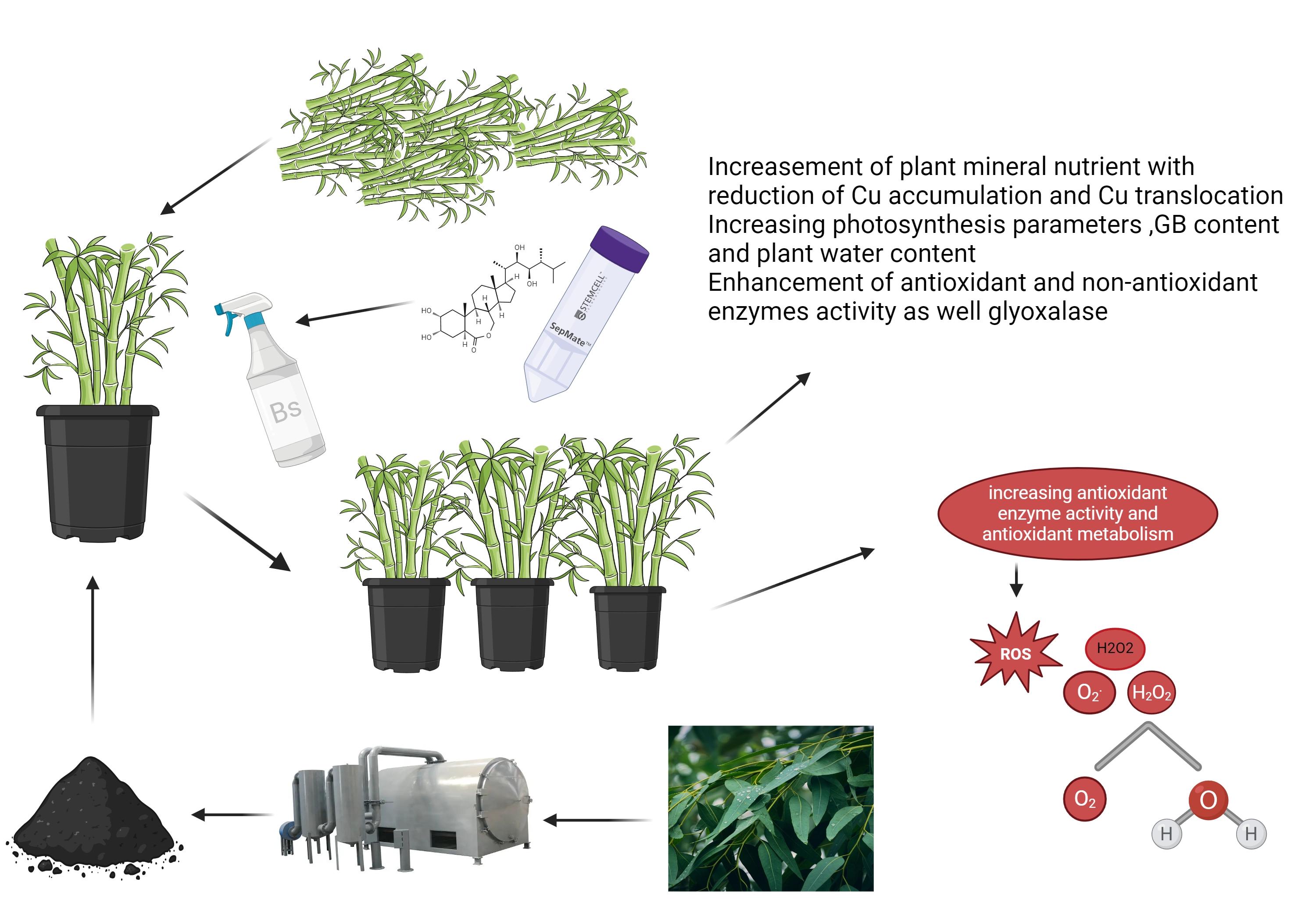

Supplement: Supplementary file 2 [file Image1.tiff]
